# Treatments for the Fifth Metacarpal Neck Fractures: A Network Meta-analysis of Randomized Controlled Trials

**DOI:** 10.1097/MD.0000000000003059

**Published:** 2016-03-18

**Authors:** Shuang-Le Zong, Gang Zhao, Li-Xin Su, Wei-Dong Liang, Li-Geng Li, Guang Cheng, Ai-Jun Wang, Xiao-Qiang Cao, Qiu-Tao Zheng, Li-Dong Li, Shi-Lian Kan

**Affiliations:** From the Department of Orthopedics Institute, The Second Hospital of Tangshan (S-LZ, GZ, L-XS, W-DL, L-GL, A-JW, X-QC, Q-TZ, L-DL); The Trauma Laboratory of the North China University of Science and Technology, Tangshan, Hebei Province (GC); and Department of Orthopedics Institute, Tianjin hospital, Tianjin Medical University, Tianjin, People's Republic of China (S-LK).

## Abstract

The fifth metacarpal neck fractures (commonly termed boxer's fractures) are the most common type of metacarpal fractures. Many types of treatments are available in clinical practice, some of which have already been compared with other treatments by various researchers. However, a comprehensive treatment comparison is lacking. We estimated the comparative efficacy of different interventions for total complications, through a network meta-analysis of randomized controlled trials.

We conducted a systematic search of the literature through October 2015. The outcome measurements were the total complications. We used a Bayesian network meta-analysis to combine direct and indirect evidence and to estimate the relative effects of treatment.

We identified 6 RCTs registering a total of 288 patients who were eligible for our network meta-analysis. The literature's quality is relatively high. The median Structured Effectiveness for Quality Evaluation of Study score for the included trials was 33.8. The overall methodological quality was high. Of the 6 studies, all were 2-arm controlled trials comparing active intervention. Among the 4 treatments—conservative treatment (CT), antegrade intramedullary nailing (AIMN), transverse pinning (TP) with K-wires, and plate fixation (PF)—CT had the best rankings (ie, lowest risk of total complications), followed by PF, AIMN, and TP (ie, highest risk of total complications). Furthermore, we also presented the results using surface under the cumulative ranking curve. The surface under the cumulative ranking curve probabilities were 94.1%, 52.9%, 37.3%, and 15.7% for CT, PF, AIMN, and TP, respectively.

In conclusion, current evidence suggested that conservative treatment is the optimum treatment for the fifth metacarpal neck fractures because of reduced total complication rates. Moreover, the TP with K-wires is the worst option with highly total complication rates. PF and AIMN therapy should be considered as the first-line choices. Larger and higher-quality randomized controlled trials are required to confirm these conclusions and better inform clinical decision-making.

## INTRODUCTION

The fifth metacarpal neck fractures (commonly termed boxer's fractures) are the most common type of metacarpal fractures,^1,2^ reported as 25% of all metacarpal fractures. Of all fractures of the upper extremity, little finger metacarpal neck fractures constitute 5% of the overall total.^3^ The fifth metacarpal neck fractures are generally associated with aggressive behaviors; these fractures are typically caused by a longitudinal compression force to the knuckles when the hand is in a clenched fist posture.^4^

The management of these fractures is still a matter of debate.^5–7^ The majority of fifth metacarpal neck fractures are isolated injuries, simple, closed, and stable, which are usually recommended for conservative treatment. Numerous indications for operative treatment include malrotation, angulation, and longitudinal shortening. In the latest decades, the operative management choice and metalwork products for the fifth metacarpal neck fractures were increased as the surgical technology and internal fixation products developed. The reasons for surgeons to decide for open or closed reduction and internal fixation also included the improvement of materials and instruments, and better understanding of biomechanical principles of internal fixation.^8^

Despite the fact that the current recommendations to treat these injuries are highly variable, a comprehensive systematic review of all approaches to treat the fifth metacarpal neck fracture does not exist. Previous systematic reviews have compared antegrade intramedullary nailing to other surgical modalities via meta-analysis.^9^ However, there are few randomized controlled trials (RCTs) comparing different active treatment strategies, which can inform clinicians and patients regarding the comparative effectiveness of these interventions. Pairwise meta-analyses provide only partial information in this case because they can only answer questions about pairs of treatments. In our systematic review, we performed a Bayesian network meta-analysis (NMA),^10–12^ combining direct and indirect evidence comparing the relative efficacy of all interventions (conservative treatment [CT], antegrade intramedullary nailing [AIMN], transverse pinning [TP] with K-wires, and plate fixation [PF]) for the treatment of fifth metacarpal neck fractures, to synthesize comprehensively the available evidence for total complications in patients, even if no studies directly compare them.

## METHODS

We carried out the NMA following the PRISMA (Preferred Reporting Items for Systematic Reviews and Meta-Analyses) Statement, which was established to help authors report a wide array of systematic reviews.^13^ Since this study was a review of published studies, ethical approval was not required, which is consistent with previous research.^14^

### Search Strategy

We searched PubMed, ScienceDirect, and Wiley online library for relevant RCTs until the end of October 2015. The restriction for English language was applied. All searches were conducted using the following retrieve tactics: (metacarpal neck fracture) AND random∗. Meanwhile, reference lists of the relevant articles were also retrieved for any additional relevant studies.

### Selection Criteria and Assessment of Methodological Quality

We systematically reviewed the studies according to the following criteria: adults older than 18 years; patients with acute, closed, little finger metacarpal neck fractures; evaluation of the RCTs of at least 2 medical interventions; reported results of postoperative complications. A pathological fracture and concomitant hand fractures were excluded from the study, and the comparison of 2 different conservative treatments was excluded.

The quality of the studies was evaluated using the Structured Effectiveness for Quality Evaluation of Study (SEQES), as seen in Table 1. The SEQES is a 24-item critical appraisal tool developed by MacDermid^15^ and used to evaluate the methodological characteristics of a study. The SEQES was used to rate the quality of the article methodology by a standardized form. The score sheet contains 7 general sorts: study question, study design, subjects, intervention, outcomes, analysis, and recommendations. The SEQES score sheet includes 24 items, and each item is given a score of 2, 1, or 0. The maximum total score that can be achieved is 48. A quality score between 33 and 48 was considered to indicate high quality, scores between 17 and 32 indicated moderate quality, and scores ≤16 indicated low quality. Each of the reviewer's SEQES scores was blinded to the other reviewer until the scores were compared. Any discrepancies in the score were discussed until a consensus was reached.

**TABLE 1:**
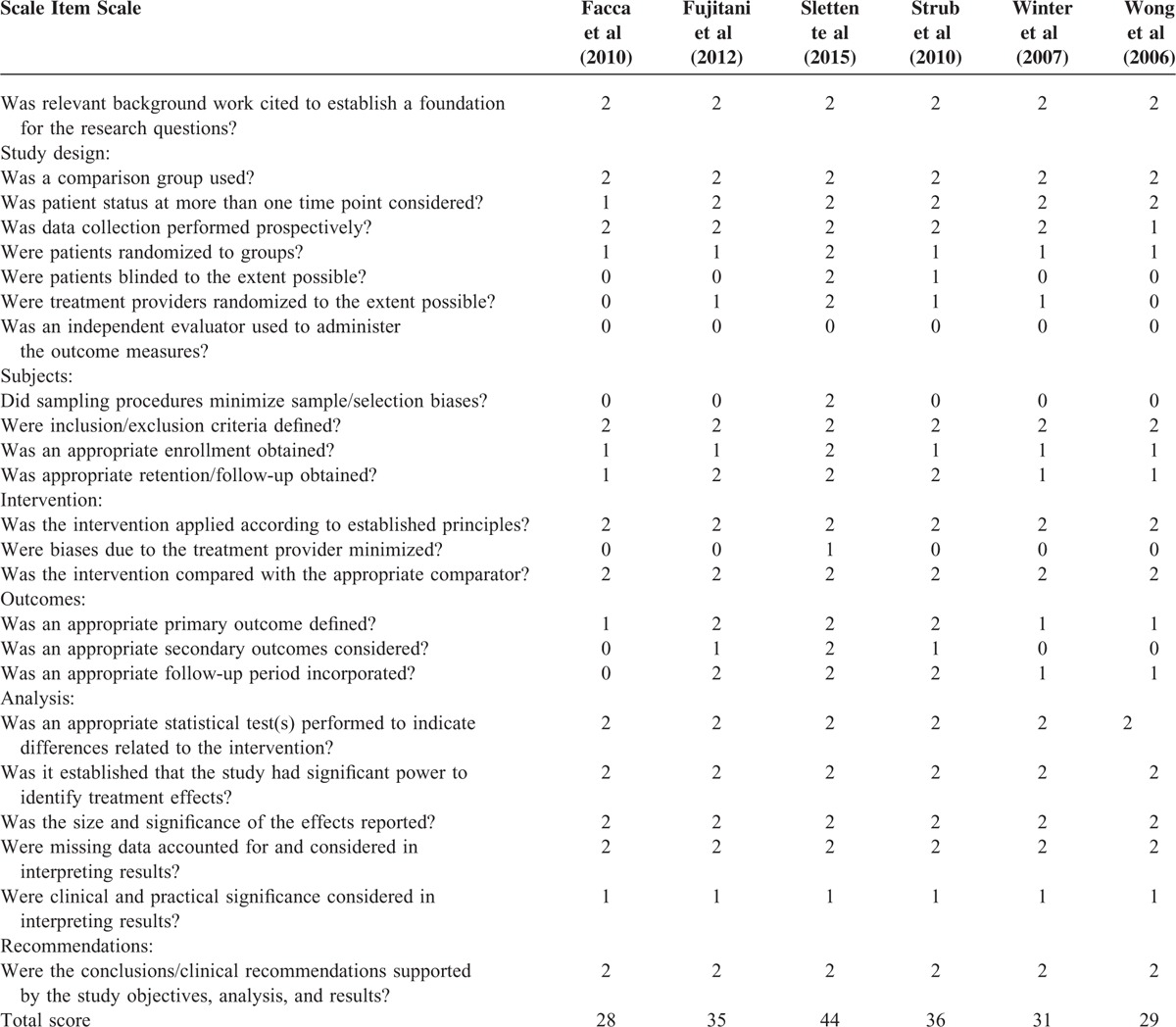
Structured Effectiveness Quality Evaluation Scale (SEQES) Scores of the Included Studies

### Data Extraction

Two of authors (S-LZ and L-XS) independently conducted the data extraction from the qualified articles. The following data were extracted: random method, patient characteristics, interventions, and patient-based outcomes. The primary outcome was the incidence of total complications. The extracted data were re-examined by another author (S-LK).

### Data Synthesis and Analysis

Our NMA was conducted using WinBUGS (version 1.4.3, MRC Biostatistics Unit, Cambridge, UK), with random-effects network described by Chaimani et al.^16^ In our NMA, the posterior statistical parameters were performed by Markov chain Monte Carlo methods.^17^ Then, we used the graphical tools in STATA12 (StataCorp, TX) to present the results of statistical analyses of WinBUGS1.4.3. Noninformative uniform and normal prior distributions were performed to fit the model.^18^ An automatically generated starting value was used to fit the model. We updated Markov chain Monte Carlo model with 11,000 simulated draws after a burn in of 1000 iterations. The total complications were presented as odd ratios (ORs) with 95% confidence intervals (CIs). To rank the treatments, we used the surface under the cumulative ranking probabilities (SUCRA) to indicate which treatment was the best one.^19^ Then, the rank probability data were imported into STATA, which then produced SUCRA curve.^20^ We assessed the publication bias by using a funnel plot of the reported primary outcome.

## RESULTS

### Study Characteristics

**FIGURE 1:**
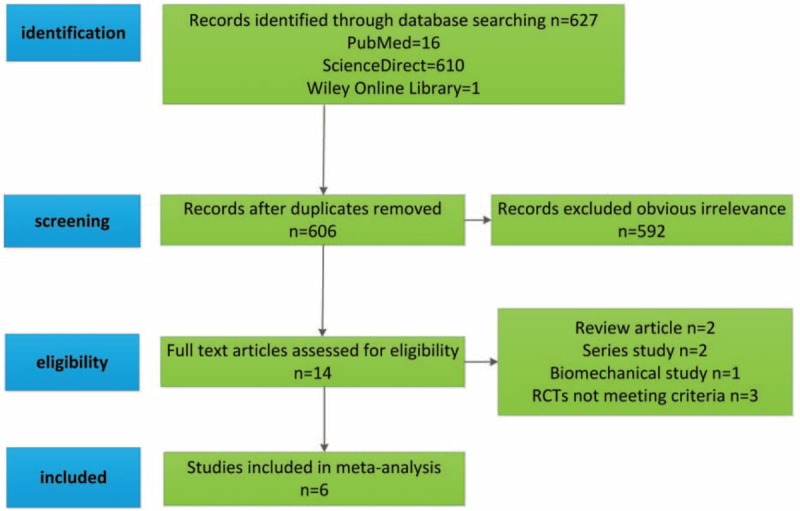
Flow chart depicting the selection process.

From the selected database, we identified a total of 627 citations as potentially relevant studies. By screening the title and reading the abstract and the full article, 6 studies eventually satisfied our eligible criteria. We found that 6 RCTs,^21–26^ registering a total of 288 patients, were eligible for our NMA. The literature search procedure was shown in Figure 1. We presented the included patients’ characteristics in Table 2.

**TABLE 2:**
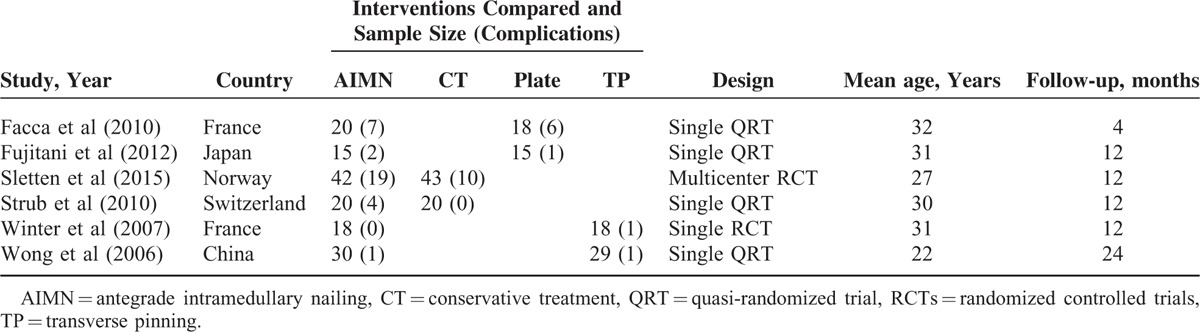
Characteristics of the 6 Included Studies

### Methodological Quality

The literature's quality is relatively high. The median SEQES score for the included trials was 33.8. The overall methodological quality was high. Of the 6 studies,^21–26^ all were 2-arm controlled trials comparing active intervention (2 comparing AIMN with CT, 2 comparing AIMN with PF, 2 comparing AIMN with TP). One trial reported adequate allocation concealment and blinding of patients and study personnel. The detailed SEQES quality scores of the included studies are summarized in Table 1.

#### Network Configuration

**FIGURE 2:**
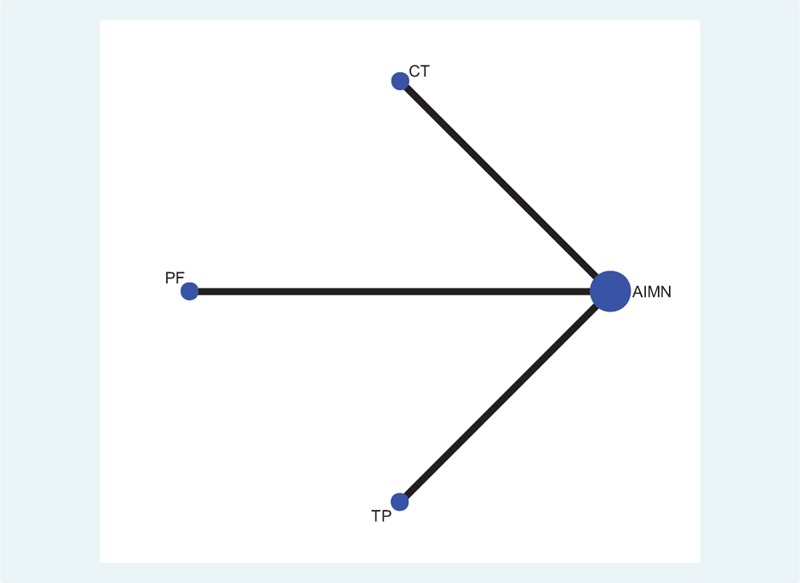
Network of the comparisons for the Bayesian network meta-analysis. Note: the size of the circle represents the number of patients, and the line width reflects the number of direct comparisons. No connecting line between 2 treatments indicates that there was no direct comparison. AIMN = antegrade intramedullary nailing, CT = conservative treatment, PF = plate fixation, TP = transverse pinning.

Comparisons between treatments and number of studies for each contrast were shown in Figure 2. The network configuration shows direct comparisons. The other comparisons without connecting lines can be compared indirectly through Bayesian NMA. The size of nodes symbolizes the total sample size of treatments. The thickness of lines symbolizes the number of trials. Four treatments were included in our NMA for the fifth metacarpal neck fractures.

#### Contribution Configuration of Network Meta-analysis

**FIGURE 3:**
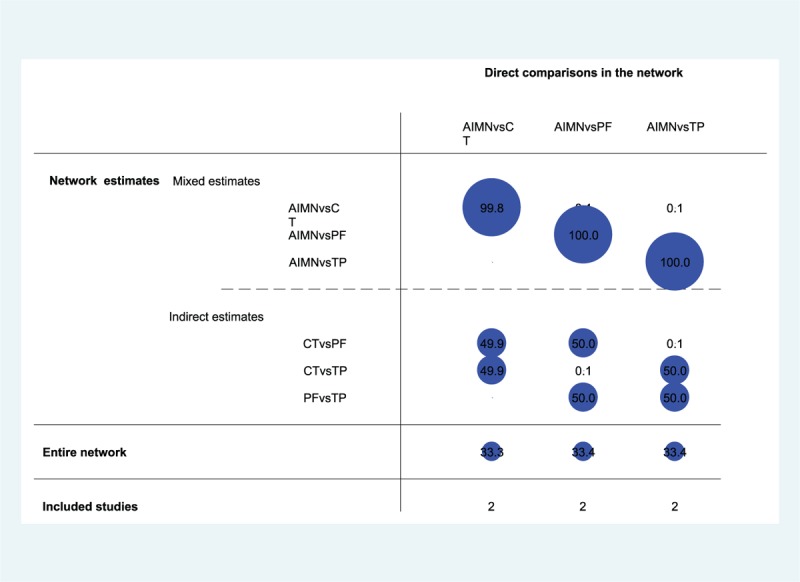
Contribution plot of enrolled studies in this network meta-analysis. Note: the size of the circle represents the percentage contribution of each direct comparison in the network.AIMN = antegrade intramedullary nailing, CT = conservative treatment, PF = plate fixation, TP = transverse pinning.

The contribution of each study to the network summary effects is reported in Figure 3: 2 studies reported direct comparison between AIMN and CT, whose mixed estimates were 99.8%, the indirect estimates were 49.9% for CT versus PF, 49.9% for CT versus TP, and 33.3% for the entire NMA; 2 studies illustrated direct comparison between AIMN and PF, whose mixed estimates were 100%, the indirect estimates were 50.0% for CT versus PF, 50.0% for PF versus TP, and 33.4% for the entire NMA; 2 studies demonstrated direct comparison between AIMN and TP, whose mixed estimates were 100%, the indirect estimates were 50.0% for CT versus TP, 50.0% for PF versus TP, and 33.4% for the entire NMA.

#### Comparison of Total Complications Rate

**FIGURE 4:**
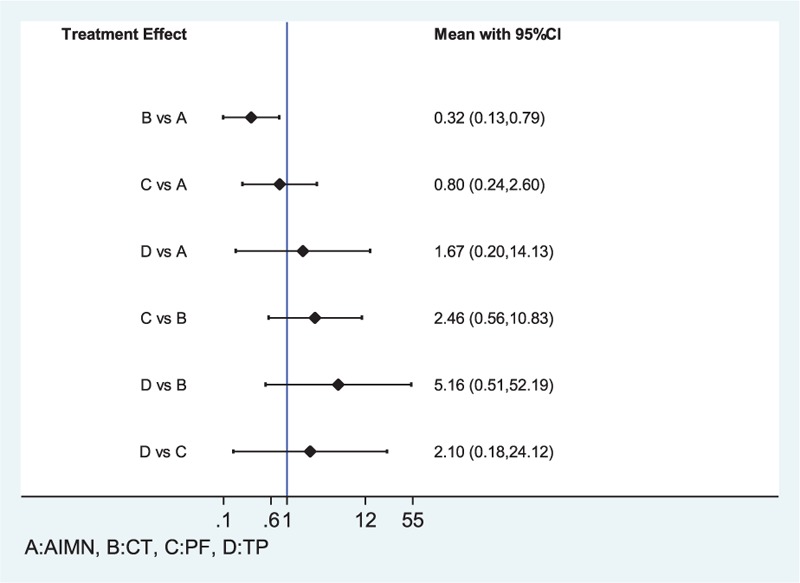
Treatments compared with each other in total complications. Note: the blue line represents no effect line. Statistically significant effect sizes were found only between the CT and AIMN interventions. AIMN = antegrade intramedullary nailing, CI = confidence interval, CT = conservative treatment, PF = plate fixation, TP = transverse pinning.

The NMA results showed that the only comparison between CT and AIMN had significantly different total complications rate (OR 0.32; 95% CI, 0.13–0.79). However, no significant difference was found in the total complications rates between the other treatments for the fifth metacarpal neck fractures (Figure 4).

#### Network Meta-analysis of the Total Complications

Data on rate of total complications was reported in all 6 studies.^21–26^ The total complication contains infection, complex regional pain syndrome, carpal tunnel syndrome, refracture, neurological injury, tendon injury, loss of reduction, additional surgery to removal hardware, pin migrated and revision.

**FIGURE 5:**
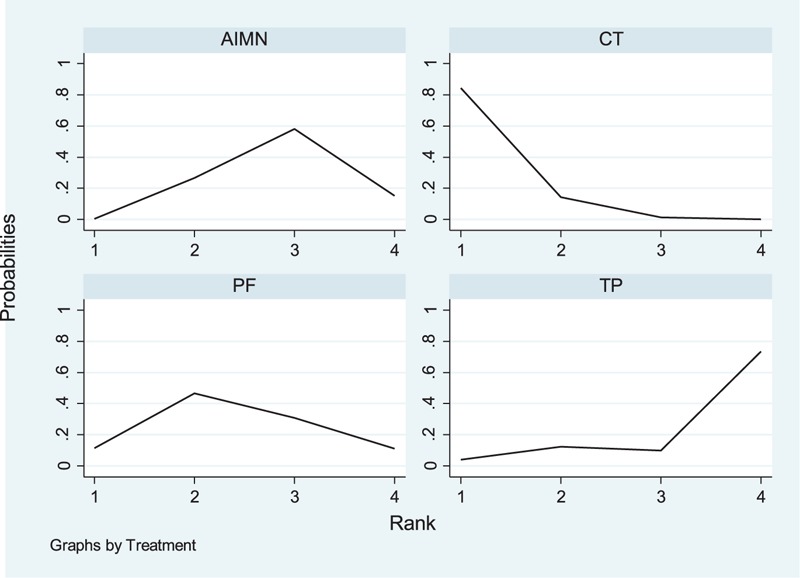
The rankograms probability of inpatients with the fifth metacarpal neck fractures treated with 4 treatments. Note: the value of the horizontal axis corresponding to the curve's peak represents the cumulative rank of each treatment. AIMN = antegrade intramedullary nailing, CT = conservative treatment, PF = plate fixation, TP = transverse pinning.

**FIGURE 6:**
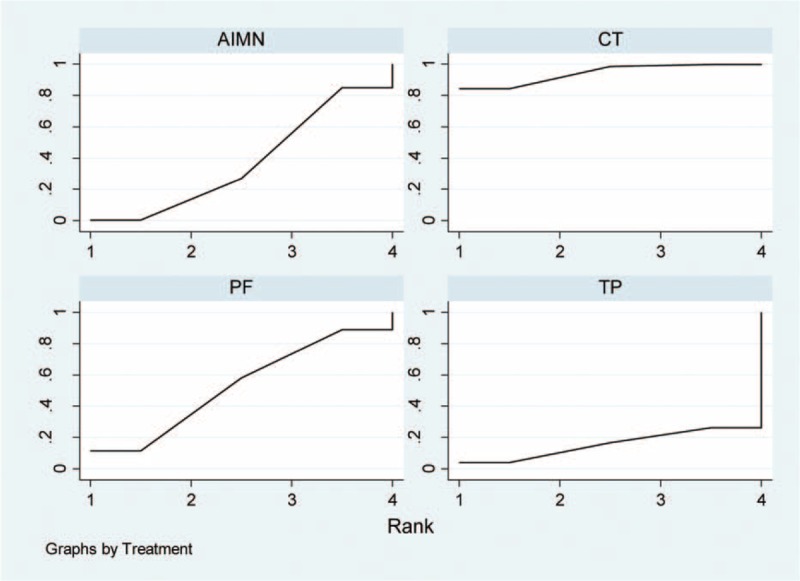
Surface under the cumulative ranking curves of treatment relative ranking of predictive probabilities for comparisons of the total complications in patients with the fifth metacarpal neck fractures treated with 4 treatments. Note: the area under the curve represents the cumulative rank probability of each treatment, with larger areas signifying higher probabilities. AIMN = antegrade intramedullary nailing, CT = conservative treatment, PF = plate fixation, TP = transverse pinning.

Rankograms probability indicated the possibility of each treatment being the best, the second best, and so forth down to the worst treatment. The information on relative rankings (along with the probabilistic analysis) represents the most interesting result of our analysis (Figure 5). Among the 4 treatments, CT had the best rankings (ie, lowest risk of total complications), followed by PF, AIMN, and TP (ie, highest risk of total complications). Furthermore, we also presented the results using SUCRA. The SUCRA probabilities were 94.1%, 52.9%, 37.3%, and 15.7% for CT, PF, AIMN and TP, respectively (Figure 6). A higher SUCRA value suggested better results for respective treatment modality.

#### Publication Bias

**FIGURE 7:**
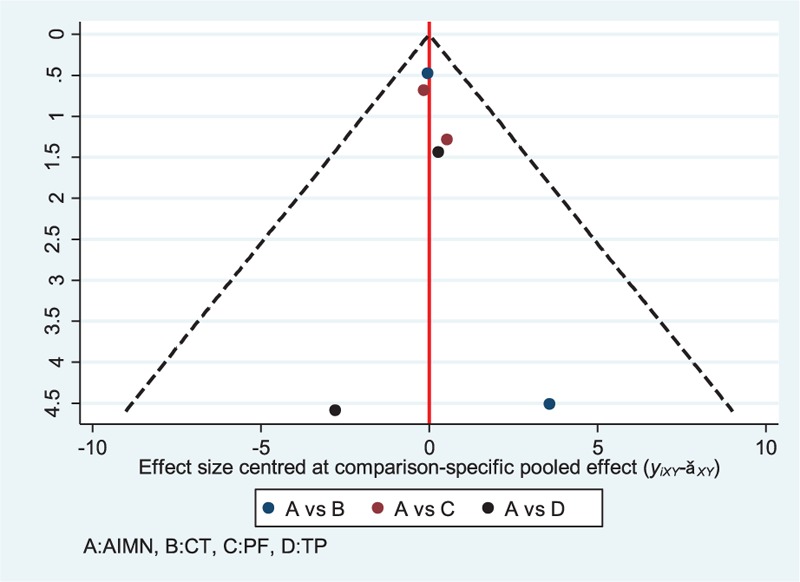
Funnel plot of the Bayesian network meta-analysis for assessing publication bias in all six included studies. Note: symmetry of the plot can indicate no publication bias. AIMN = antegrade intramedullary nailing, CT = conservative treatment, PF = plate fixation, TP = transverse pinning.

A funnel plot of total complications was established to evaluate publication bias (Figure 7). In Figure 7, the funnel plot is symmetrical to the line and it implies that there are no small study effects in our NMA. Although the number of studies included in each comparison was very small, making the available methods for evaluating publication bias is unreliable.

## DISCUSSION

To our knowledge, there are no large studies comparing all intervention types for the fifth metacarpal neck fractures, and a NMA offers opportunities to make comparisons that otherwise do not exist in head-to-head comparative trials. Our results provided a synthesis of the effectiveness data of the main widely used treatments for the fifth metacarpal neck fractures. It was successful in determining the statistical significant differences between all treatments and in defining their respective rankings. We therefore aimed to use a NMA to determine the optimal option with the lowest risk of total complications.

The management of fractures of the fifth metacarpal neck continues to be highly variable because it is difficult to discern from the literature the optimal management of these injuries. Traditionally, the fifth metacarpal neck fractures have been treated with closed reduction and immobilization, but they tend to dislocate easily after reduction.^27^ In our NMA, we support the conservative treatment with less total complications rate. However, Strub et al^23^ suggested that the residual dorsal angulation, patient satisfaction, and the aesthetic results seem to be better in the operational group than in the conservative one. Surgical treatment benefits the manual workers with a more rapid recovery, but with a relative high risk for total complications. However, there was no evidence-based clinical practice guideline recommending one form of treatment over another. Therefore, we believe that treatment options should be chosen on individual basis. Conservative treatment makes this cost-effective management worthwhile, even in the presence of deformity. To date, it remains disputable how much dorsal displacement can be tolerated. Several clinical studies have reported that in the conservative treatment of the fifth metacarpal neck fractures (casting, with or without reduction) between 20° and 70° of residual dorsal angulation is acceptable.^28–30^ Many surgeons generally regard that angulation of 30° is an indication for surgery.

In our NMA, we found that the plate fixation had less total complications rate than AIMN, but there was no significant differences (OR 0.80; 95% CI, 0.24–2.60). Moreover, the CIs for these 2 treatment comparisons overlapped with “no effect,” therefore were not precise enough to be confident regarding the direction of effect. In the surgical treatment, we recommend the AIMN and PF for better treatment. However, from a cost-analysis perspective, the cost of PF was considered to be much higher than that of K-wire fixation. And Facca et al^26^ reported the plate fixation treatment developed a serious complication of the fifth metacarpal head necrosis. So we preferred the AIMN in certain circumstances. TP with K-wires showed a higher risk of total complications compared with other surgical treatment options in our NMA (ie, plate fixation and AIMN). As a result, the TP with K-wires is not recommended.

We could not quantify differences in the risks of single complication among the comparisons; small numbers of reported events prevented us from finding any appreciable differences between treatment options. Other clinically relevant outcomes such as grip strength, range of motion, pain, and patient satisfaction were not examined as they were inconsistently reported. This may limit the utility of the study somewhat in any future meta-analyses. Future randomized trials must clearly report the detailed data of mean and standard deviation.

Our findings add important new information to the existing literature. There has been 1 systematic review^9^ using traditional head-to-head meta-analyses for the fifth metacarpal neck fractures’ surgical management, which suggests that the AIMN technique could have some advantages over the use of mini-plate fixation or transverse pinning in terms of clinical outcomes in patients with the fifth metacarpal neck fractures. But that systematic review does not contain the conservative treatment. Our NMA provides clarity regarding conclusions that can be made based on the current literature.

The strengths of our analyses included the comprehensive assessment of the relative efficacy of all available treatments for total complications in the fifth metacarpal neck fractures. It should be acknowledged that we have included all the randomized controlled studies and therefore there is no possibility to have “more evidence” than that provided in this study. However, there were certain limitations in our studies. First, only 6 RCTs^21–26^ were incorporated in our study to compare 4 treatments for the fifth metacarpal neck fractures, and the number of included studies and sample size were relatively small. Second, although all the selected RCTs mentioned the design of randomized control, blinding method, a detailed evidence of randomization was lacking. Furthermore, we cannot completely rule out publication bias; higher efficacy is more commonly found in smaller trials. Methodological limitations such as small sample sizes, randomizations, and total follow-up all contribute to heterogeneity in published complication rates and should be taken into account when interpreting these data. So our results must be interpreted with caution.

## CONCLUSIONS

In conclusion, current evidence suggests that conservative treatment is the optimum treatment for the fifth metacarpal neck fractures because of reduced total complication rates. And TP with K-wires is the worst option with highly total complication rates. Among the surgical treatments, PF and AIMN therapy should be considered as the first-line choices. Unfortunately, based on the results of our NMA, it remains difficult for clinicians to make informed decisions about which treatment is the best surgical option (the plate fixation or the AIMN). Larger and higher-quality RCTs are required to confirm these conclusions and better inform clinical decision-making.
